# COVID-19 als Versicherungsfall der gesetzlichen Unfallversicherung: Berufskrankheit oder Arbeitsunfall: Relevantes Wissen für den (Allgemein‑/Viszeral‑)Chirurgen

**DOI:** 10.1007/s00104-023-01892-z

**Published:** 2023-06-02

**Authors:** Irina Böckelmann, Frank Meyer, Beatrice Thielmann

**Affiliations:** 1 Bereich Arbeitsmedizin, Medizinische Fakultät, Otto-von-Guericke-Universität Magdeburg, Magdeburg, Deutschland; 2 Universitätsklinik für Allgemein-, Viszeral-, Gefäß- und Transplantationschirurgie, Otto-von-Guericke-Universität Magdeburg mit Universitätsklinikum Magdeburg A. ö. R., Magdeburg, Deutschland

**Keywords:** SARS-CoV-2, Pandemie, Versicherungsfall, Berufsgenossenschaft (BG), Unfallkasse, Deutsche Gesetzliche Unfallversicherung, SARS-CoV-2, Pandemic, Insured events, Professional disease insurance company, Accident insurance company, German Lawful Accident Insurance (“Deutsche Gesetzliche Unfallversicherung“)

## Abstract

Die SARS-CoV-2-Pandemie führte zu vielen Infektionen mit dem Virus und Erkrankungen an Coronavirus-Krankheit-2019 (COVID-19). Als Konsequenz davon war ein enormer Anstieg gemeldeter und anerkannter Berufskrankheiten (BK) und Arbeitsunfälle (AU) bei den Berufsgenossenschaften (BG) und Unfallkassen als Träger der Deutschen Gesetzlichen Unfallversicherung zu verzeichnen. Die Publikation hat das Ziel, die Unterschiede von BK oder AU aufzuzeigen und die aktuellen Daten zum BK-Geschehen aufzuarbeiten. Dabei wird auf Definitionen von BK und AU, die Unterschiede in den Voraussetzungen für eine Anerkennung im Sinne einer BK oder eines AU eingegangen. Des Weiteren werden die Leistungsansprüche dargestellt. Zuletzt werden statistischen Kennzahlen der BK nach Nr. 3101 und den AU dargestellt.

Ergebnisse (Eckpunkte):

– AU und BK sind nach § 7 SGB VII Versicherungsfälle der gesetzlichen Unfallversicherung.

– In der Chirurgie wird wie spezifisch im übrigen Gesundheitswesen die Relevanz der SARS-CoV-2-Infektion mit ihrer Post-COVID beim Personal für die arbeitsmedizinische Vorsorge und als anzuerkennender Fall der gesetzlichen Unfallversicherung (BK oder AU) deutlich.

– Maßgeblich für die Anerkennung sind die Dauer und die Intensität des Kontaktes (örtliche Nähe) – die SARS-CoV-2-Arbeitsschutzregel vom 20.08.2020 sieht hier im Wesentlichen eine Kontaktdauer von mindestens 15 min bei einer räumlichen Entfernung von weniger als 1,5–2 m vor (weitere Aspekte: intensiverer kürzerer Kontakt, Anzahl der nachweislich infizierten Personen im engeren Tätigkeitsumfeld bzw. der üblichen Personenkontakte, räumliche Situation, Arbeitsweg, besondere Konstellationen).

– Für die detaillierte Darstellung des Settings Chirurgie können keine Fallzahlen eruiert werden. – Bei der Begutachtung von COVID-19-Folgen bzw. „Post-COVID“ als BK bestehen noch immense Probleme und Herausforderungen, da zahlreiche Unsicherheitsfaktoren wie z. B. unzureichend gesichertes Wissen zum weiteren Langzeitverlauf über die Jahre oder das breit gefächerte Symptomspektrum die ärztliche Beurteilung der Folgen dieser Erkrankung erschweren.

Schlussfolgerung: Die SARS-CoV-2-Pandemie stellt sich als eine besondere Herausforderung der Chirurgie mit fachspezifisch immanent intensiverem Patientenkontakt bzw. des gesamten Gesundheitswesens dar, die durchaus langanhaltende Veränderungen verursachte und deren adäquate gesundheitsbetreuerische wie auch versicherungsrechtliche Aufarbeitung der (fallspezifischen) Konsequenzen noch beträchtliche Anstrengungen und Ressourcen erfordern dürfte.

## Abstract (en)

The severe acute respiratory syndrome coronavirus 2 (SARS-CoV-2) pandemic resulted in many infections with the virus and sickness due to coronavirus disease 2019 (COVID-19). Therefore, there was a dramatic increase in the number of reported and recognized occupational diseases (*Berufskrankheit*, BK) and occupational accidents (*Arbeitsunfall*, AU) at the German Social Accident Insurance Institutions (BG) and accident insurance funds (*Unfallkassen*).

The publication aims to show the differences between BK and AU and to review the current data on occupational diseases. It deals with the definitions of BK and AU, the differences in the conditions for recognition as BK or AU. Furthermore, the claims for benefits are presented. Finally, statistical key figures of the BK according to No. 3101 and the AU are presented.

Results (key points)

– According to § 7 SGB VII, AU and BK are insured events of the statutory accident insurance.

– In surgery, like specifically in the rest of the healthcare system, the relevance of the SARS-CoV‑2 infection with post-COVID in personnel for occupational medical prevention and as a case to be recognized by the statutory accident insurance (BK or AU) becomes clear.

– Relevant for the recognition are the duration and the intensity of the contact (local proximity) and the SARS-CoV‑2 occupational health and safety rule of 20 August 2020 essentially recognizes a contact duration of at least 15 min at a spatial distance of less than 1.5–2 m (further aspects: more intensive shorter contacts, number of verifiably infected persons in the closer activity environment or the usual personal contacts, spatial situation, work route, special constellations).

– No case numbers can be elicited for the detailed presentation of the surgery setting.

– There are still immense problems and challenges in the assessment of COVID-19 consequences or post-COVID as occupational disease, as numerous uncertainty factors, such as insufficiently secured knowledge about the further long-term course over the years or the widely varied symptom spectrum complicates the medical assessment of the consequences of this disease.

Conclusion: the SARS-CoV‑2 pandemic is a special challenge for surgery with intensive patient contact and for the entire healthcare system. This caused long-lasting changes and the adequate health care as well as insurance law processing of the (case-specific) consequences might still require considerable efforts and resources.

## Einführung

Die rasche Verbreitung des SARS-CoV‑2 („severe acute respiratory syndrome coronavirus 2“) führte am 11.03.2020 zur Erklärung einer weltweiten pandemischen Lage durch die Weltgesundheitsorganisation [1]. Zu den als bekannt vorausgesetzten beruflichen Risiken im Gesundheitswesen kam eine neue Infektion durch virale Krankheitserreger hinzu. Beschäftigte im Gesundheitsbereich haben im Vergleich zu Beschäftigen aus anderen Branchen ein 2,1-fach erhöhtes Risiko für eine SARS-CoV-2-Infektion [2]. Die Tab. 1 bietet einen Steckbrief des SARS-CoV-2-Virus nach [3].

**Table 1:** 

*Erreger*	„Severe acute respiratory syndrome coronavirus type-2“ (SARS-CoV‑2; Coronaviridae, Genus Betacoronavirus, Subgenus Sarbecovirus)
*Erkrankung*	Covid-19 (Coronavirus 19, „corona virus disease 19“)
*Struktur*	Coronaviren plus Hämagglutinin-Esterase Durchmesser 80–140 nm Einzelstrang-RNA Spike-Protein (S-Protein) für den Eintritt in die Wirtszelle – bindet an „angiotensin converting enzyme 2“ (ACE2) sowie an Neuropilin‑1 Spike-Protein induziert neutralisierende Antikörper → Entwicklung von Impfstoffen
*Übertragung*	Respiratorische Aufnahme von Tröpfchen oder Aerosolen – Schmierinfektion nicht auszuschließen
*Infektiosität*	Hohe Kontagiosität, v. a. zum Zeitpunkt des Symptombeginns
*Varianten*	Bedeutendeste: Alpha B.1.1.7, Beta B.1.351, Gamma P.1, Delta B.1.617.2 und Omikron B.1.1529
*Nachweis*	Ag-Schnelltest PCR-Test (Polymerasekettenreaktion-Test) als Goldstandard RT-LAMP („reverse transcription-loop mediated amplication“) NAT (Nukleinsäureamplikationstechniken)
*CT-Wert*	Wertebereich 1–40 Niedriger Wert bedeutet hohe Viruslast

Die Chirurgie stand plötzlich vor tiefgreifenden Umgestaltungen der Arbeitsprozesse und veränderter Arbeitsorganisation [4–6]. Chirurgisches Personal hat ein hohes arbeitsbedingtes Risiko für eine Infektion mit dem SARS-CoV‑2. Bei Ausübung der Tätigkeit kann es zum direkten Kontakt mit einem wahrscheinlich oder bestätigt infektiösen Patienten kommen. Es wurden über 28 Mio. chirurgische Operationen verschoben [7], wobei die dringlichen oder lebenserhaltenden Operationen an Coronavirus-Krankheit-2019(COVID-19)-Patienten weiter durchgeführt wurden [4, 8]. Mitarbeiter der chirurgischen Fachdisziplinen können auch selbst eine SARS-CoV-2-Infektion auf Patienten übertragen. Zeitnahe Anpassungen an die neue Arbeitssituation, sichere chirurgische Versorgung von Patienten, Reduzierung des Risikos bei dem von Anbeginn einer erhöhten Infektionsgefährdung ausgesetzten Personal, arbeitsschutzkonformer Umgang mit COVID-19-Patienten und persönliche Schutzausrüstung (PSA) sind Entwicklungen und Antworten, die die Pandemie von den Verantwortlichen in der Chirurgie in kurzer Zeit verlangte. Positiv ist, dass ersten Studienergebnissen zufolge die zusätzliche PSA bei chirurgischen Eingriffen, während einer COVID-19-Pandemie keine relevanten Auswirkungen auf die geistige und körperliche Leistungsfähigkeit des Chirurgen hatte. Hier bedarf es allerdings weiterer Studien [9]. Neben den Auswirkungen der Pandemie auf die chirurgische Versorgung allgemein ist die Problematik des fehlenden chirurgischen Personals infolge einer eigenen Infektionskrankheit oder durch Quarantäne sowie der Herausforderungen für die eigene Gesundheit nach einer stattgehabten Infektion ein weiteres wichtigeres Problemfeld.

Die ersten Monate der Pandemie verliefen in Ungewissheit, es fehlten evidenzbasierte Empfehlungen der Fachgesellschaften, es mangelte an Kenntnissen der Ausbreitung des SARS-CoV‑2 und der Risikoeinschätzungen, die Folgen der COVID-19 waren nicht vorhersehbar. Klassische Regeln der Erkenntnisbewertung durch die wissenschaftliche Gemeinschaft und die Regeln der evidenzbasierten Medizin fanden nur begrenzt Anwendung [8, 10]. Um das Risiko der Selbstkontamination beim Ablegen der persönlichen Schutzausrüstung zu minimieren, mussten Beschäftigte der chirurgischen Fachdisziplinen geschult und trainiert werden [8, 10]. Die Zahl des infizierten Personals im Gesundheitswesen war sehr hoch. Bei einem Teil davon wurde die Diagnose „Long- bzw. Post-COVID-19-Syndrom“ dokumentiert [11]. Nach drei Jahren besonderer Herausforderungen steht fest: Auch in der Chirurgie wird die Relevanz der SARS-CoV-2-Infektion mit ihrer Post-COVID beim chirurgischen Personal für die arbeitsmedizinische Vorsorge und als ein anzuerkennender Fall der gesetzlichen Unfallversicherung (Berufskrankheit [BK] oder Arbeitsunfall [AU]) deutlich.

Diese Publikation beschäftigt sich mit den arbeitsmedizinischen Fragen, um das ausreichende Wissen der Ärzteschaft in der Chirurgie über mögliche Zusammenhänge von beruflich bedingtem Risiko gegenüber einer SARS-CoV-2-Infektion während der chirurgischen Tätigkeiten und möglichen arbeitsbedingten Erkrankungen des Personals zu vermitteln. Außerdem sollen die wichtigsten Grundlagen für die Erstellung einer Anzeige einer BK nach Nr. 3101 der Anlage zur BK-Verordnung bei begründetem Verdacht oder einer Meldung eines AU im Falle einer Infektion mit dem SARS-CoV‑2 sowie die aktuellen Daten zum BK-Geschehen in Deutschland wiedergegeben werden. Basiskenntnisse über BK sind für jeden Chirurgen und Mediziner erforderlich (wie bereits hinreichend durch die Arbeitsmedizin im Rahmen des Humanmedizinstudiums vermittelt).

## Ergebnisse (Eckpunkte)

### Definition der Versicherungsfälle

AU und BK sind nach § 7 SGB VII Versicherungsfälle, gegen die eine Absicherung über die gesetzliche Unfallversicherung besteht [12].

#### Definition Berufskrankheit

Der Versicherungsfall Berufskrankheit (BK) wird im § 9 des SGB VII aufgeführt [12]. „Anerkennung einer Berufskrankheit“ betrachtet man eher als einen Rechtsbegriff und nicht als medizinischen Terminus, welcher durch Rechtsverordnung mit Zustimmung des Bundesrates als BK bezeichnet wird [13]. Auf der Seite des Bundesministeriums für Arbeit und Soziales (BMAS) findet man die Definition der Berufskrankheiten: „Erkrankungen, die Versicherte durch ihre berufliche Tätigkeit erleiden und die in der Berufskrankheiten-Verordnung (BKV) aufgeführt sind“ [14]. Des Weiteren gilt für die BK „die nach den Erkenntnissen der medizinischen Wissenschaft durch besondere Einwirkungen verursacht sind, denen bestimmte Personengruppen durch ihre versicherte Tätigkeit in erheblich höherem Grade als die übrige Bevölkerung ausgesetzt sind“ [12]. Das gilt insbesondere für BK Nr. 3101: „Infektionskrankheiten, wenn der Versicherte im Gesundheitsdienst, in der Wohlfahrtspflege oder in einem Laboratorium tätig oder durch eine andere Tätigkeit der Infektionsgefahr in ähnlichem Maße besonders ausgesetzt war“ [15].

#### Definition Arbeitsunfall

Der Versicherungsfall AU wird im § 8 des SGB VII aufgeführt. Als AU von Versicherten wird ein „zeitlich begrenztes, von außen auf den Körper einwirkendes, unfreiwilliges, schädigendes Ereignis, das mit einer versicherten Tätigkeit in ursächlichem Zusammenhang steht und eine Gesundheitsschädigung oder den Tod bewirkt hat.“ verstanden [12].

### Meldepflicht der Versicherungsfälle und Infektionskrankheiten in Deutschland

#### Meldepflicht einer Berufskrankheit gemäß § 202 SGB VII

Jeder Arzt und Zahnarzt ist nach § 202 SGB VII verpflichtet, den begründeten Verdacht auf das Vorliegen einer BK, die auch in der Anlage der BK-Verordnung gelistet ist [16], bei dem zuständigen Träger der Gesetzlichen Unfallversicherung (GUV) oder dem Staatlichen Gewerbearzt bzw. Landesgewerbearzt anzuzeigen [17]. Neben den Ärzten besteht für Unternehmer Anzeigepflicht, wenn die Anhaltspunkte für eine mögliche BK vorliegen. Des Weiteren können auch gesetzlich Unfallversicherte, Krankenkassen, Rentenversicherungsträger, Arbeitsamt u. a. den Verdacht auf das Vorliegen einer BK anzeigen. Dafür gibt es ein amtliches „Formular für die ärztliche Anzeige bei Verdacht einer BK“ [18].

#### Meldepflicht eines Arbeitsunfalls gemäß § 193 SGB VII

Eine gesetzliche Meldepflicht für den Arbeitgeber besteht für AU im Betrieb, durch die versicherte Personen getötet oder so verletzt worden sind, wobei sie mehr als drei Tage arbeitsunfähig sind (§ 193 SGB VII). Die Frist von drei Tagen beginnt am Tag nach dem Unfall und umfasst alle Kalendertage [19].

#### Meldepflicht gemäß Infektionsschutzgesetzes

Jeder behandelnde Arzt nach § 6 „Meldepflichtige Krankheiten“ des Infektionsschutzgesetz (IfSG) sowie jedes diagnostizierende Labor nach § 7 „Meldepflichtige Nachweise von Krankheitserregern“ des IfSG müssen meldepflichtige Krankheiten, Verdachtsfälle einer Erkrankung, die Erkrankungen, den Tod sowie Labornachweise auf eine akute Infektion an das zuständige Gesundheitsamt melden [20]. Das betrifft auch die Infektionen mit dem SARS-CoV-2-Virus und meldepflichtige COVID-19-Erkrankungen.

#### Übermittlung der Daten zur COVID-19 über die zuständige Landesbehörde an das RKI

Das Gesundheitsamt übermittelt die Daten zur meldepflichtigen COVID-19-Erkrankung und zu laborbestätigten COVID-19-Fällen an das Robert Koch-Institut (RKI), die in der Pandemiezeit täglich einen aktuellen Lagebericht zu COVID-19 veröffentlicht [21]. Donnerstags erscheint ein ausführlicher Wochenbericht zur COVID-19-Lage.

Zwar kann man in der RKI-Statistik die Daten differenziert nach Tätigkeit in Einrichtungen mit besonderer Relevanz für die Transmission betrachten (z. B. § 23 IfSG [z. B. Krankenhäuser, ärztliche Praxen] und § 36 IfSG [z. B. Pflegeeinrichtungen]), jedoch wird bei diesen Meldungen nach IfSG und den Daten des Lageberichts nicht unterschieden, ob es sich um die außerberuflich erworbene Infektionen (Ansteckung im privaten Bereich) oder um arbeitsbedingte Infektionen (bedingt durch die Infektionsgefährdung am Arbeitsplatz) handelt und ob es um medizinisch oder nichtmedizinisch tätiges Personal geht. Das ist aber für die Anerkennung der COVID-19 als BK nach dem Merkblatt zur Berufskrankheit Nr. 3101 entscheidend sowie für die Prävention und den Infektionsschutz am Arbeitsplatz essenziell [22].

### Versicherungsfälle der gesetzlichen Unfallversicherung

#### Berufskrankheit Nr. 3101

Bei BK Nr. 3101 der Anlage zur Berufskrankheiten-Verordnung (BKB) handelt es sich um Infektionskrankheiten, wenn der Versicherte im Gesundheitsdienst, in der Wohlfahrtspflege oder in einem Laboratorium tätig oder durch eine andere berufliche Tätigkeit der Infektionsgefahr in ähnlichem Maße besonders ausgesetzt war. Der Ärztliche Sachverständigenbeirat „Berufskrankheiten“ beim Bundesministerium für Arbeit und Soziales (BMAS) prüft orientierend im Vorfeld, welche Krankheiten nach epidemiologischen, infektiologischen und pathophysiologischen Erkenntnissen entsprechend der Definition des BK-Begriffs zur Aufnahme in die Liste der BK vorgeschlagen werden und welche Tätigkeitsfelder identifiziert werden können, bei denen ein versichertes (berufliches) vergleichbar hohes Infektionsrisiko wie bei den Beschäftigten im Gesundheitswesen besteht. Diese Krankheiten werden von Mensch zu Mensch übertragen, wie es bei COVID-19-Erkrankung auch der Fall ist.

#### COVID-19 als Arbeitsunfall

Erfolgt eine Infektion mit SARS-CoV‑2, ohne dass die Voraussetzungen einer BK erfüllt sind, kann die COVID-19-Erkrankung einen AU darstellen. COVID-19 kann auch als ein AU anerkannt werden, wenn die Erkrankten versicherte Tätigkeiten außerhalb des Gesundheitswesens ausüben und wenn sich der intensive und direkte Kontakt zu mit SARS-CoV-2-infizierten Personen nicht bestimmungsgemäß wie bei der BK 3101, sondern sonst situativ aus der Tätigkeit ergibt.

Der zuständige Träger der GUV prüft im Einzelfall, ob die Voraussetzungen zur Anerkennung einer COVID-19-Erkrankung als BK oder als AU erfüllt sind.

**Figure 1:**
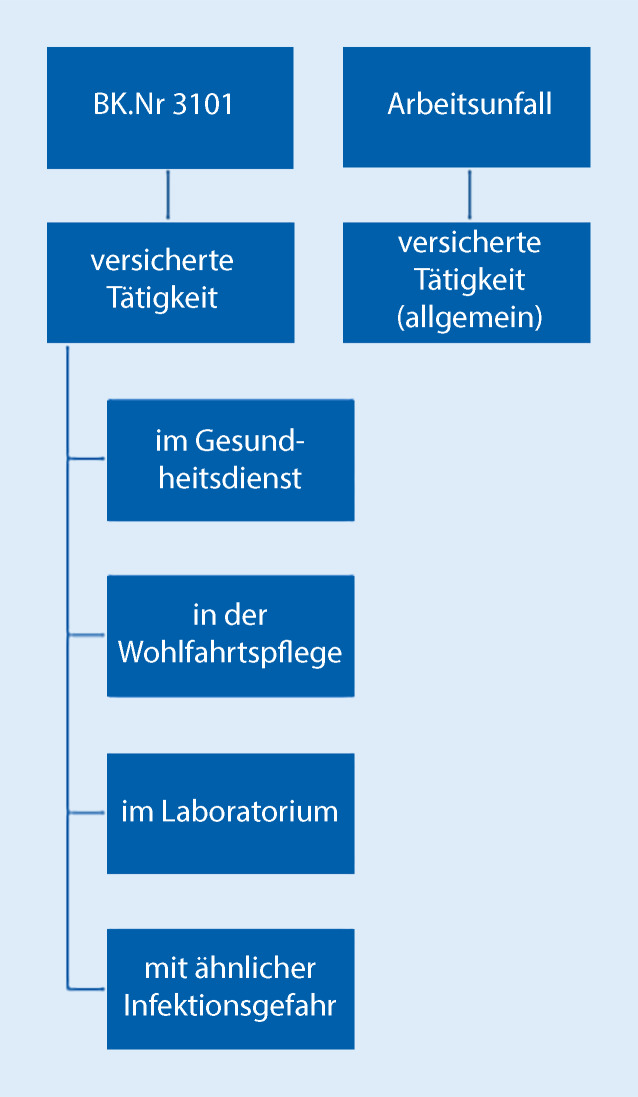

Die Abb. 1 und die Literatur lassen erkennen, wann zwischen BK oder AU im Allgemeinen unterschieden wird [23]. Somit sind bei Chirurgen eher eine Anzeige auf die BK nach Nr. 3101 als auf AU zu erwarten. Die Informationen zu den AU werden hier ergänzend dargestellt.

Im weiteren Verlauf werden die Voraussetzungen für die Anerkennung nach BK 3101 und als AU detaillierter dargestellt.

#### Voraussetzungen der Anerkennung BK 3101

Im Sozialrecht der GUV gilt das Kausalitätsprinzip für die Anerkennung einer BK: Haftungsbegründete und haftungsausfüllende Kausalität muss nachgewiesen sein. Es wird im Allgemeinen ein Vollbeweis für die versicherte Tätigkeit, die schädigende Einwirkung und den Gesundheitsschaden gefordert.

Zwischen der versicherten Tätigkeit und der schädigenden Einwirkung muss ein ursächlicher Zusammenhang (haftungsbegründete Kausalität) nachgewiesen werden. Im Fall der BK 3101 muss eine zeitliche Verbindung zwischen Exposition gegenüber dem SARS-CoV‑2 (z. B. Kontakt mit SARS-CoV-2-infizierten Patienten, Kollegen, Besuchern oder biologischem Material im Gesundheitswesen im Rahmen der beruflichen Tätigkeit) und der Infektion bestätigt werden, dabei muss man die Angaben zur Inkubationszeit beachten. Die Infektion muss ebenfalls im Vollbeweis nachgewiesen werden, z. B. durch Labornachweise von SARS-CoV‑2 im positiven PCR-Test vom diagnostizierenden Labor. Gerade in der Anfangsphase der Pandemie fehlten bei vielen Erkrankten die Labornachweise, weil die Testungen nicht durchgeführt wurden. Die BGW weist darauf hin, dass der Verdacht auf das Vorliegen der BK Nr. 3101 bei einer im Gesundheitsdienst tätigen versicherten Person auch in der anderen Konstellation begründet ist: beim nicht vorliegenden positiven PCR(polymerase chain reaction)-Test bei einer versicherten Person bei Ausübung ihrer versicherten Tätigkeit mit einem direkten Kontakt zu einer wahrscheinlich oder bestätigt mit SARS-CoV‑2 infizierten Person, wenn bei dieser versicherten Person nach diesem Kontakt innerhalb der Inkubationszeit Symptome einer COVID-19-Erkrankung (Müdigkeit/Erschöpfung, Kopfschmerzen, Gliederschmerzen, Verlust des Geschmacks‑/Geruchssinns, Husten, Konzentrations‑/Gedächtnisprobleme, Kurzatmigkeit, Halsschmerzen, Schnupfen, Fieber u. ä.) auftreten [24]. Als ein direkter Kontakt wird insbesondere bei pflegerischer Tätigkeit oder körperlicher Untersuchung oder beim Umgang mit Körperflüssigkeiten angesehen [24].

Zwischen der schädigenden Einwirkung und der Erkrankung muss ein wahrscheinlicher Zusammenhang bestehen, d. h. der kausale Zusammenhang zwischen Exposition gegenüber dem SARS-CoV‑2 und COVID-19 (Krankheitssymptome wie Fieber, Husten usw.) muss nicht zwangsläufig im Vollbeweis nachgewiesen werden, sondern er muss wahrscheinlich sein. In diesem Fall genügen auch geringfügige klinische Symptome, d. h. sie müssen nicht zwangsläufig so ausgeprägt sein, dass damit eine ärztliche Behandlungsbedürftigkeit oder die Notwendigkeit einer medikamentösen Behandlung besteht. Hier reicht Wahrscheinlichkeit („mehr spricht dafür als dagegen“) für die gutachterliche „Bejahung“ des Kausalzusammenhangs zwischen schädigender beruflicher Einwirkung und Gesundheitsschaden aus. In diesem Fall spricht man von einer haftungsausfüllenden Kausalität.

Der Nachweis ist erbracht, wenn vor dem Hintergrund des pandemischen Geschehens und der mitunter hohen Infektionszahlen in der Allgemeinbevölkerung die berufliche Verursachung überwiegend wahrscheinlich ist und keine Hindernisse aus dem unversicherten Bereich einer Anerkennung entgegenstehen – dann ist eine Anerkennung der BK Nr. 3101 möglich [22, 25].

Der Kausalzusammenhang liegt bei der COVID-19 als BK Nr. 3101 in der Regel vor, wenn die versicherte Person in dem infrage kommenden Ansteckungszeitraum bei ihrer versicherten Tätigkeit Kontakt zu mindestens einer nachgewiesener Infektionsquelle hatte, nach der Art des Kontaktes eine Infektionsübertragung des SARS-CoV‑2 dabei konkret möglich war und Verwicklungen aus dem außerberuflichen Bereich (z. B. privat Kontakt im Haushalt einer an SARS-CoV-2-Infektion erkrankten Person, bei Freizeitaktivitäten oder im Urlaub) einem Schluss auf die Wahrscheinlichkeit des Zusammenhangs mit der versicherten Tätigkeit nicht entgegenstehen [25].

Für die Wahrscheinlichkeit der für eine Kausalität ausreichenden Gefahrerhöhung bei COVID-19 ist zwischen der Verbreitung der Infektionskrankheit und dem Übertragungsweg zu unterscheiden, dabei sind einige Tätigkeiten/Bereiche zu nennen, die zu einer Beweiserleichterung führen können. Dazu gehören Klinikabteilungen und -stationen, in denen Patienten mit COVID-19 behandelt werden, intensivmedizinische Behandlungseinheiten, Notfallintubation u. Ä.

**Figure 2:**
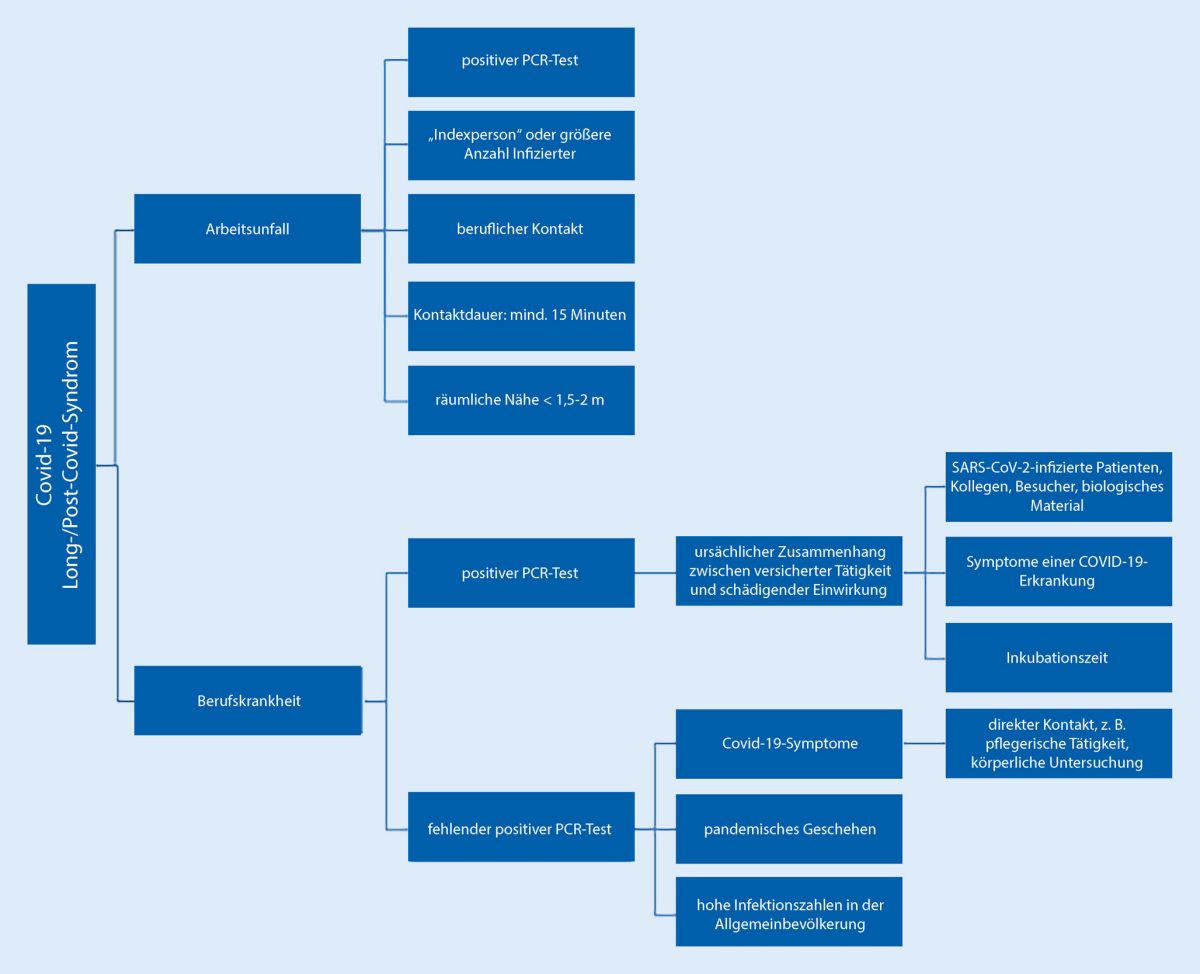

Abb. 2 stellt eine Zusammenfassung der Bedingungen für eine BK oder einen AU dar.

#### Voraussetzungen der Anerkennung COVID-19 als Arbeitsunfall

Die COVID-19-Infektion muss auf eine nachweislich infizierte Person („Indexperson“) mit dem Labornachweis von SARS-CoV‑2 im positiven PCR-Test vom diagnostizierenden Labor zurückzuführen sein. Der intensive berufliche Kontakt mit dieser Indexperson muss bestanden haben und nachgewiesen werden. Die Erkrankung muss spätestens innerhalb von zwei Wochen nach dem Kontakt eingetreten sein.

Im Einzelfall, wenn kein intensiver Kontakt zu einer Indexperson festzustellen war, kann ausreichend sein, um die berufliche Verursachung infolge der versicherten Tätigkeit nachzuweisen, wenn es eine größere Zahl nachweislich infektiöser Personen im Betrieb gegeben hat und konkrete, die Infektion begünstigende Bedingungen bei der versicherten Tätigkeit vorgelegen haben [26].

Maßgeblich für die Anerkennung sind die Dauer und die Intensität des Kontaktes (örtliche Nähe). Die SARS-CoV-2-Arbeitsschutzregel vom 20.08.2020 (BMAS) sieht hier eine Kontaktdauer von mindestens 15 min bei einer räumlichen Entfernung von weniger als 1,5–2 m vor [27]. Im Einzelfall kann der zeitlich kürzere Kontakt auch für die Anerkennung ausreichend sein, wenn es sich um eine besonders intensive Begegnung handelte und umgekehrt (längerer Kontakt bei dem eingehaltenen Mindestabstand; [26]).

Weitere Aspekte, die eine wichtige Rolle spielen können, sind Anzahl der nachweislich infizierten Personen im engeren Tätigkeitsumfeld, Anzahl der üblichen Personenkontakte, räumliche Situation (Belüftung, klimatische Bedingungen; [26]).

Auch eine COVID-19-Infektion auf dem Weg zur und von der Arbeit (vom Unternehmen organisierte Gruppenbeförderung, Fahrgemeinschaften) oder in bestimmten Konstellationen auch beim Kantinenbesuch oder beim Aufenthalt in Gemeinschaftsräumen und -unterkünften kann prinzipiell als Ursache für den AU dienen [25].

Zusammenfassend ist zu sagen, dass ein reiner Antikörpernachweis ohne Symptomatik keine Voraussetzung für eine BK oder einen AU darstellt [25].

#### Leistungsspektrum der gesetzlichen Unfallversicherung im Falle COVID-19 als BK Nr. 3101

Bei einer Anerkennung der COVID-19-Erkrankung des gesetzlich Unfallversicherten als BK kann es für die betroffene Person verschiedene Maßnahmen bedeuten [25]:

- *Kompensation/Entschädigung* in Form der Zahlung einer BK-Rente für die anerkannten BK-Fälle mit der bleibenden Minderung der Erwerbsfähigkeit beim Versicherten ≥ 20 %;
- *Rehabilitation* in Form der Zahlung der Leistungen der medizinischen Rehabilitation und der Unterstützung bei der beruflichen Rehabilitation (z. B. Verdienstausgleich bei Umsetzung auf einen schlechter bezahlten Arbeitsplatz oder bei Umschulung in einen anderen Beruf) sowie die Übernahme der Pflegeleistungen;
- *Prävention *in Form der Veranlassung arbeitsmedizinischer Präventionsmaßnahmen und Arbeitsschutzmaßnahmen, z. B. berufliche Umschulungsmaßnahmen.

Die Kosten der Heilbehandlung, wenn die COVID-19-Erkrankung als BK anerkannt wird, übernimmt die gesetzliche Unfallversicherung. Im Todesfall können die Hinterbliebenen der versicherten Person eine Hinterbliebenenrente erhalten.

#### Leistungsspektrum der gesetzlichen Unfallversicherung im Falle COVID-19 als Arbeitsunfall

Versicherungsrechtlich gesehen, handelt es sich hier um die gleichwertigen Versicherungsfälle wie BK Nr. 3101 in Bezug auf die Ansprüche auf das Leistungsspektrum der GUV (z. B. ärztliche Behandlung, Medikamenten- und Heilmittelversorgung, stationäre Behandlung, Rente wegen Minderung der Erwerbsfähigkeit; [25]).

### Statistik

Im Jahr vor der Pandemie 2019 hatte man zum Vergleich 80.132 Verdachtsanzeigen auf eine BK gestellt und 18.156 Fälle anerkannt [28]. Die Statistik bezieht sich auf alle BK-Verdachtsfälle gemäß SGB VII § 9. Aktuell sind in der BK-Liste 82 BK aufgeführt. Bei weiteren 17.108 wurde eine berufliche Verursachung festgestellt, d. h. Fälle, bei denen besondere versicherungsrechtliche Voraussetzungen nicht erfüllt waren. Mit Änderung des BK-Rechts ab 2021 gibt es keine berufliche Verursachung mehr ohne Erfüllung der versicherungsrechtlichen Voraussetzungen bzw. ohne Anerkennung als BK [29].

Durch Corona ist die Situation deutlich verändert: Im Jahr 2020 waren es insgesamt 106.491 Anzeigen auf Verdacht einer Berufskrankheit, davon 30.329 COVID-19-Erkrankungen und 76.162 übrige BK. Die Anzeigen auf Verdacht einer BK im Zusammenhang mit COVID-19 stellten einen Anteil von knapp 30 % Verdachtsanzeigen zu allen 82 in der BK-Liste genannten BK dar [30]. Im Jahr danach stieg die Anzahl der Anzeigen weiter: 226.611 Anzeigen auf Verdacht einer BK gesamt, davon 152.173 COVID-19-Erkrankungen. Die Anzeigen auf übrige BK ging dagegen herunter: 74.438 Anzeigen.

Die Zahlen zur aktuellen Statistik, wie viele Verdachtsmeldungen auf eine BK COVID-19 und wie viele AU-Meldungen die Unfallversicherungsträger in den drei Jahren der Pandemie erhalten haben und wie viele davon bislang anerkannt wurden, findet man auf der Homepage der Deutschen Gesetzlichen Unfallversicherung (DGUV) [31].

In dieser DGUV-Statistik sind nicht nur Fälle aus dem Gesundheitswesen enthalten. Bei der BK-Statistik sind auch die versicherten Personen aus der Wohlfahrtspflege und den Laboratorien sowie jene aus einer anderen Tätigkeit mit der in ähnlichem Maße vergleichbaren Infektionsgefahr miterfasst. Bei den statistischen Angaben der DGUV zum Versicherungsgeschehen von COVID-19 als AU kommen Fälle von den Versicherten aus vielen anderen Tätigkeiten hinzu (als Beispiel: Ansteckung während einer Gruppenbeförderung zur Baustelle).

Der Großteil der BK-Anzeigen entfällt auf die BG für Gesundheitsdienst und Wohlfahrtspflege, bei der ein größerer Anteil der Beschäftigten im Gesundheitswesen gesetzlich unfallversichert ist.

**Figure 3:**
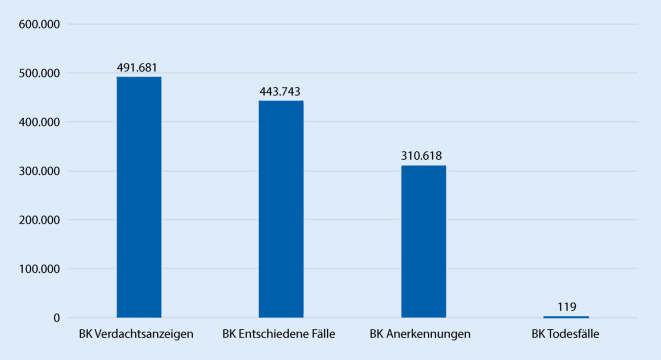

Seit Beginn der Pandemie wurden in Deutschland bei den gewerblichen BG und Unfallversicherungsträgern der öffentlichen Hand fast 500.000 Anzeigen auf Verdacht einer BK 3101 bei Versicherten mit einer COVID-19-Erkrankung gestellt (Abb. 3). Seit März 2020 hat die gesetzliche Unfallversicherung bereits in drei Viertel der Fälle COVID-19 als BK 3101 anerkannt. Insgesamt sind seit Beginn des Versicherungsgeschehens im Zusammenhang mit COVID-19 119 Todesfälle in der BK-Statistik der DGUV zu verzeichnen.

**Figure 4:**
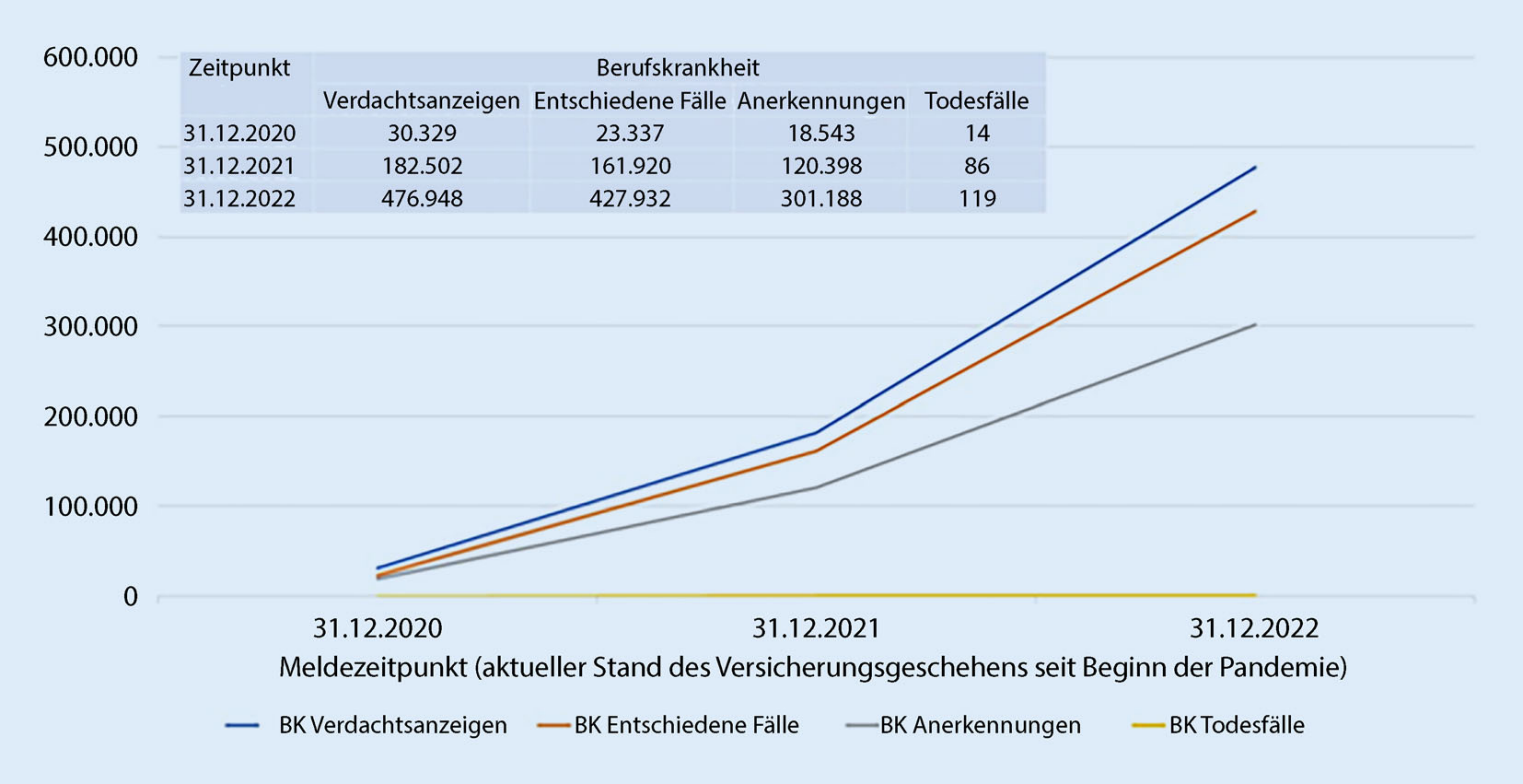

Den Verlauf des Versicherungsgeschehens (BK im Zusammenhang mit COVID-19) zu drei Meldezeitpunkten (31.12.2020, 31.12.2021 und 31.12.2022) ist in Abb. 4 dargestellt. Es ist eine deutliche Zunahme der Verdachtsanzeigen auf die BK Nr. 3101 in diesen drei Jahren zu sehen. Die Statistik der entschiedenen Fälle zeigt eine schnelle Verarbeitung der Anzeigen.

**Figure 5:**
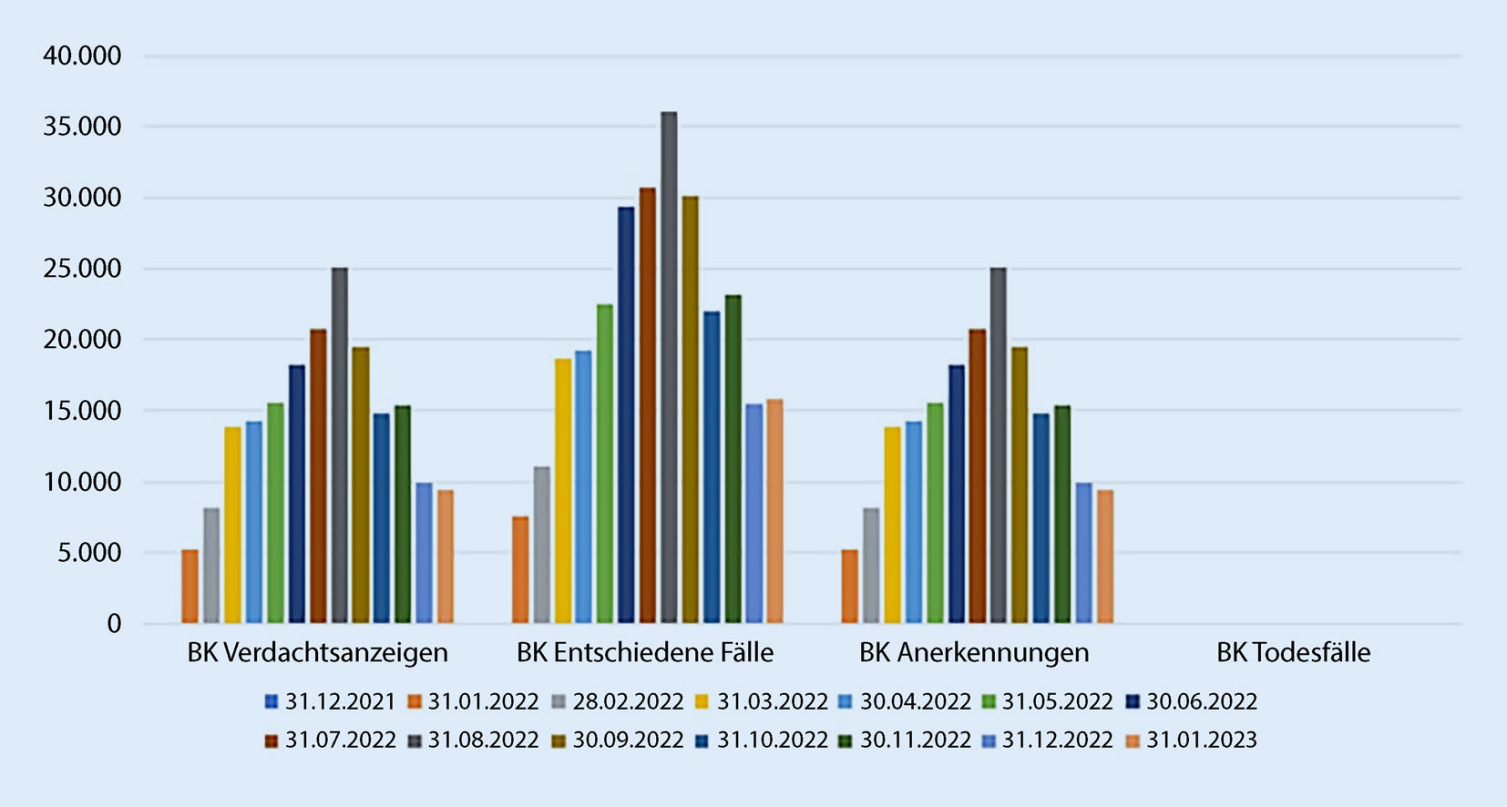

Das höchste Aufkommen der monatlich gemeldeten Verdachtsfälle war im August des dritten Jahres der Pandemie zu verzeichnen: 25.106 Verdachtsanzeigen COVID-19 als BK (Abb. 5). Im gleichen Monat schlägt auch die höchste Anzahl der entschiedenen Fälle (*n* = 36.115) und der anerkannten Fälle (*n* = 25.106) zu Buche.

**Figure 6:**
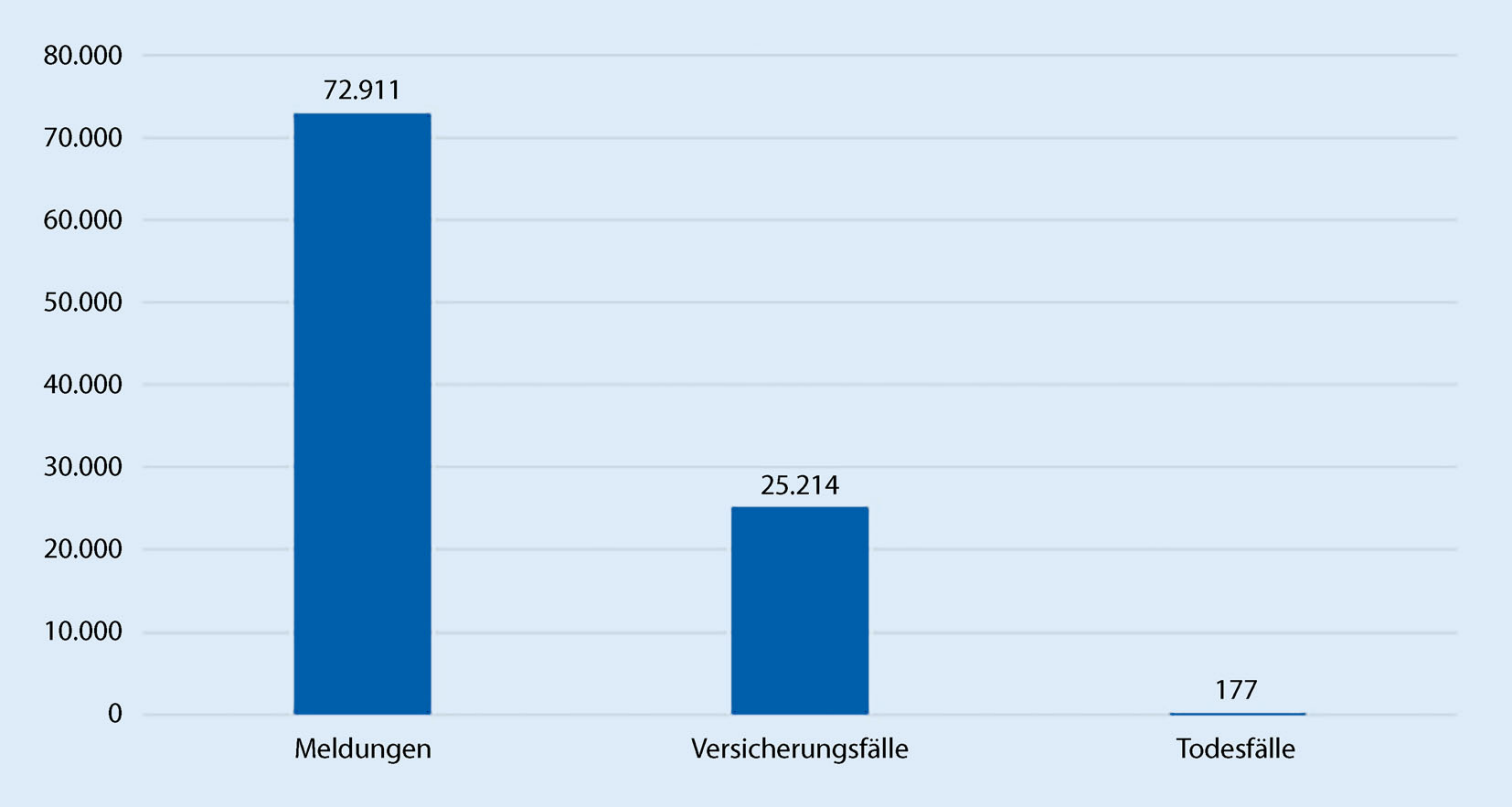

Hinzu kommen mit dem Meldezeitpunkt 31.01.2023 fast 73.000 Erkrankungen an COVID-19, die als Arbeitsunfall anerkannt wurden. Ein Drittel davon sind Versicherungsfälle (Abb. 6).

**Figure 7:**
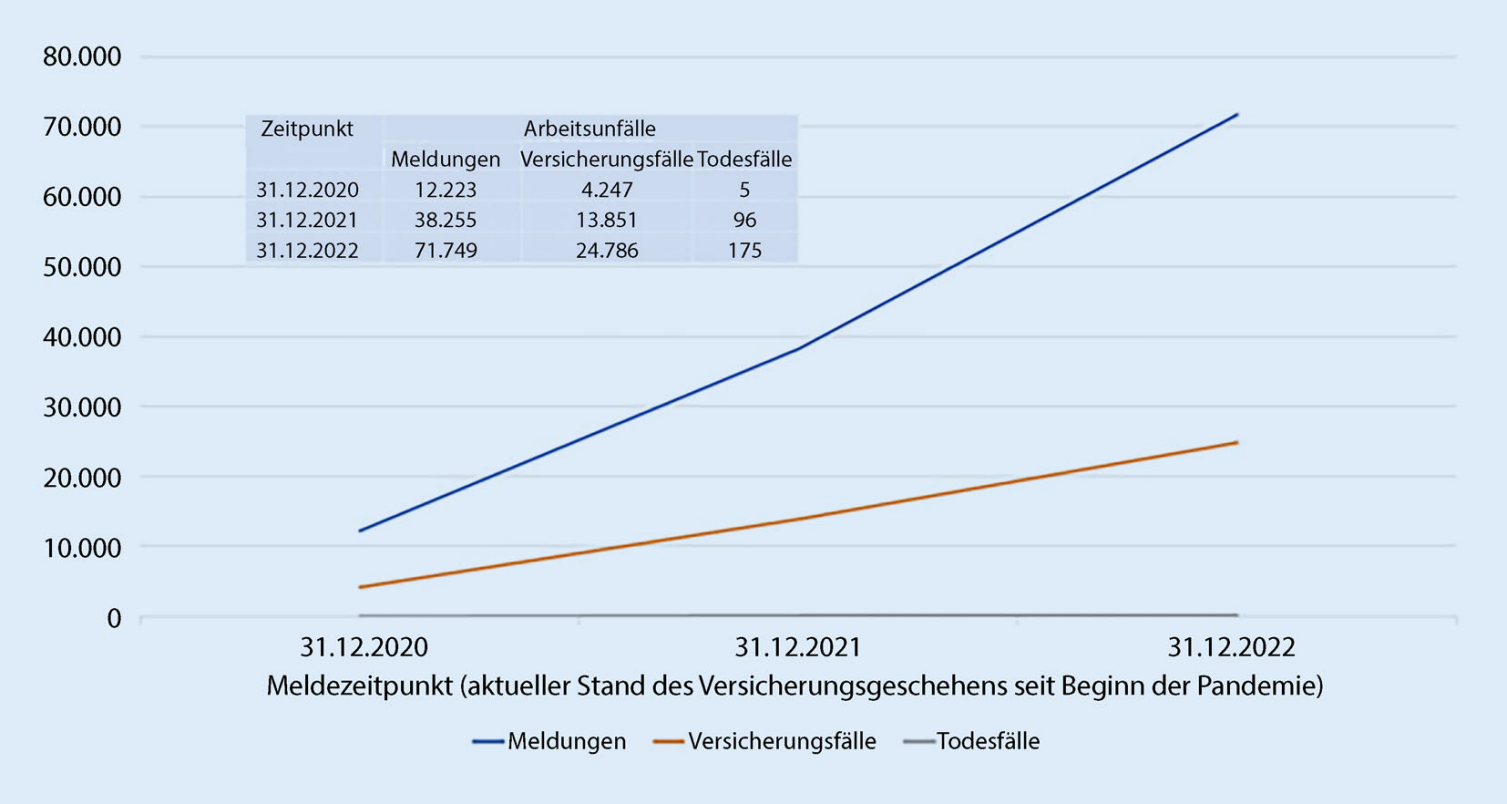

Auch bei AU im Zusammenhang mit COVID-19 sieht man eine weitere Zunahme im Versicherungsgeschehen (Abb. 7). Zum 31.12.2021 waren fast mehr als die zweifache Anzahl an Meldungen im Vergleich mit dem Meldezeitpunkt 31.12.2020 bei der Unfallversicherung im Zusammenhang mit COVID-19 als AU eingegangen: 12.223 Meldungen Ende 2020 und 26.032 neue gemeldete Fälle Ende 2021. Im Jahr 2022 kamen noch 33.494 Fälle dazu.

**Figure 8:**
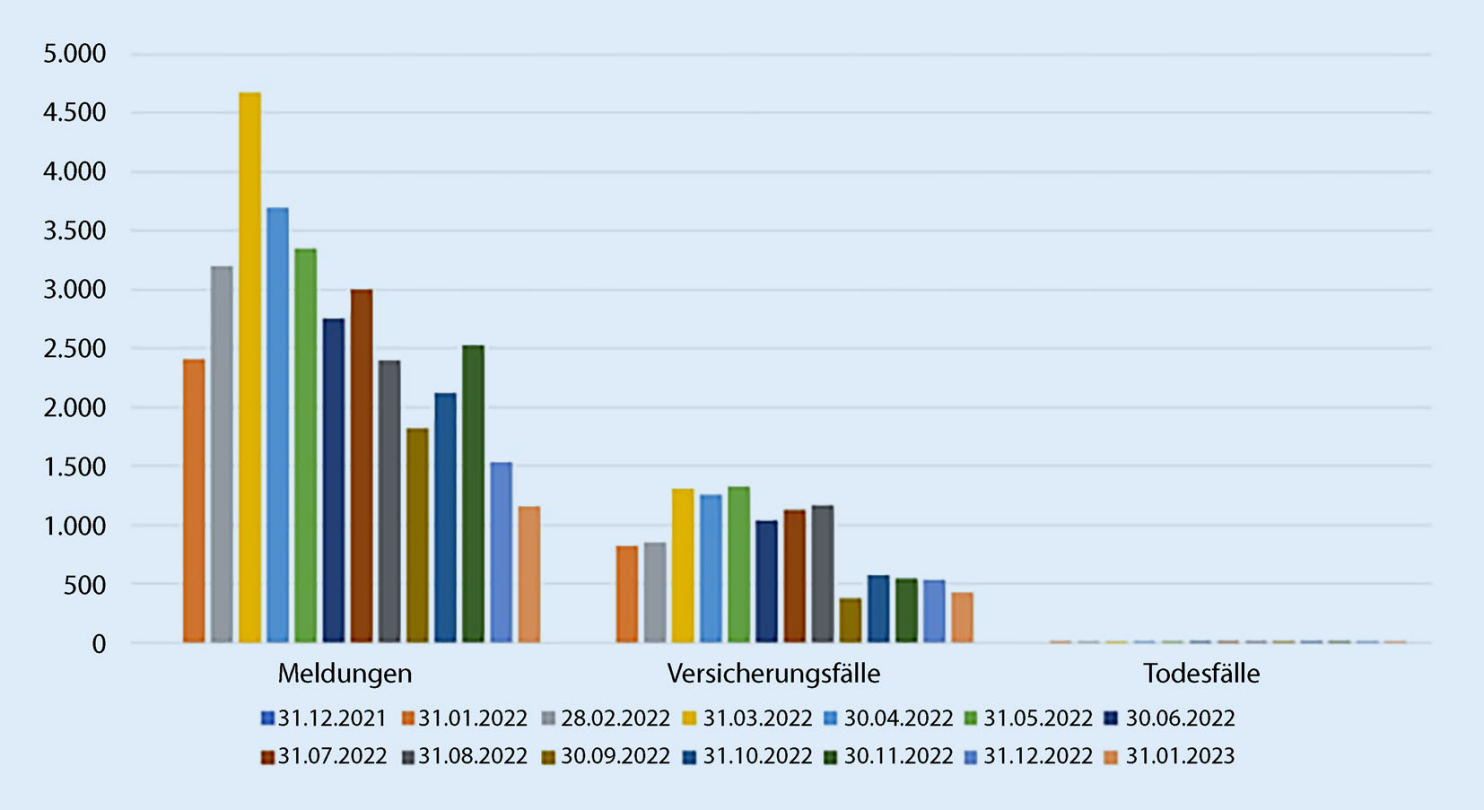

Besonders hoch war das Aufkommen der monatlichen Meldungen an COVID-19 als AU mit 4677 Meldungen im März des dritten Jahres der Pandemie (Abb. 8), gefolgt vom April 2022 mit 3692 Meldungen.

Für die Chirurgie liegen derzeit keine aktuellen Fallzahlen vor.

### Long- und Post-COVID-Syndrom als Komplikation einer COVID-19-Erkrankung

Bestehen Symptome oder treten neue, ohne andere Erkrankung als ursächlich angesehene Symptome auf, die länger als 4 Wochen postinfektiös bestehen, bezeichnet man diese Phase als eine „Long-COVID-Phase“ [11]. Die Leitlinie unterscheidet dabei

1. ein postakutes prolongiertes symptomatisches Stadium zwischen der 4. und 12. Woche (und)
2. ein „Post-COVID-Syndrom“ bei Symptompersistenz oder
  Neuauftreten von Symptomen mehr als 12 Wochen nach einer SARS-CoV-2-Infektion [11].

**Figure 9:**
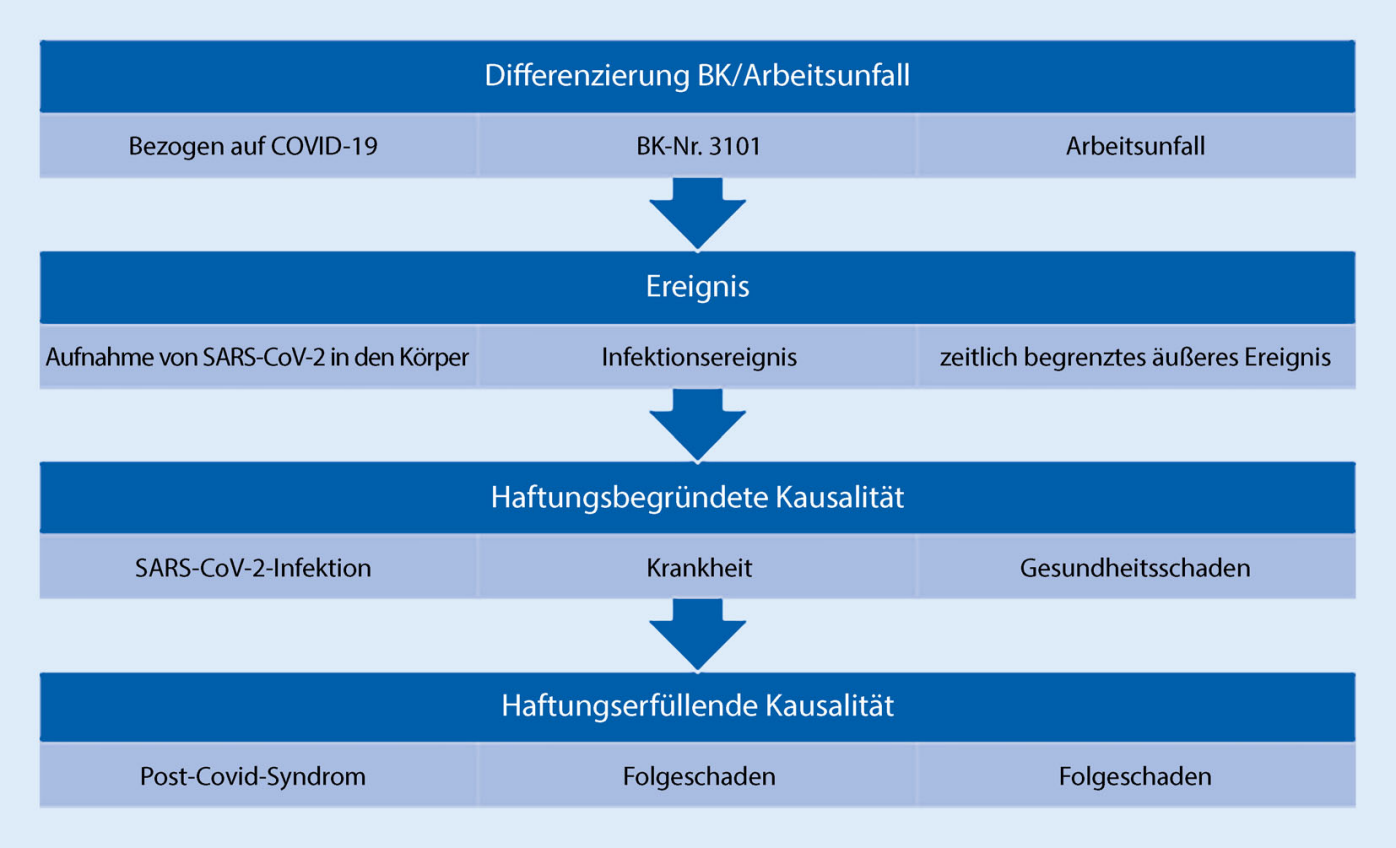

Das Post-COVID-Syndrom steht in gutachterlichen Prozessen im Fokus (Abb. 9; [23]).

Im Zusammenhang mit der Begutachtung von COVID-19-Folgen bzw. Post-COVID als BK bestehen noch Probleme und Herausforderungen, da zahlreiche Unsicherheitsfaktoren wie z. B. keinerlei Wissen zu weiterem Langzeitverlauf über die Jahre oder breites Symptomspektrum die ärztliche Beurteilung der Folgen dieser Erkrankung erschweren [28].

## Fazit

Die Tätigkeit im Gesundheitswesen während der COVID-19-Pandemie, wie die Statistik der gesetzlichen Unfallversicherung zeigt, ist mit einem deutlich erhöhten Risiko für eine SARS-CoV-2-Infektion verbunden. Der Großteil der BK-Anzeigen und AU-Meldungen kam aus dem Gesundheitsdienst.

Zurückblickend auf die Begutachtungsprozesse, muss man erwähnen, dass die gesetzliche Unfallversicherung im Zusammenhang mit der COVID-19-Begutachtung schnell reagiert und betroffenen Personen rasch umfassende medizinische, berufliche und soziale Rehabilitationsmaßnahmen ermöglicht hat.

Die Führungskräfte sind hinreichend zu schulen, um die Beschäftigten mit (überstandener) Corona-Infektion und Long-COVID bei der Rückkehr an den Arbeitsplatz zu unterstützen. Für die Beschäftigten hat die European Occupational Safety and Health Agency (EU-OSHA) einen Leitfaden für ArbeitnehmerInnen entwickelt, die nach einer Infektion mit SARS-CoV‑2 wieder an die Arbeit zurückkehren [32].

Inzwischen haben die Unfallversicherungsträger auch Versorgungsangebote für Versicherte mit Post-COVID aufgebaut wie z. B. Beratung, Sprechstunden und ein spezielles, diagnostisches Abklärungsverfahren. Die Rehabilitationsmaßnahmen werden individuell an die Bedürfnisse der Patienten angepasst und interdisziplinär durchgeführt [33]. Wissenswert ist, dass die Rückkehr in das Arbeitsleben Betroffener über eine stufenweise Wiedereingliederung meist nicht ausreichend ist. Ursächlich werden hier u. a. eine eingeschränkte Mobilität, fehlende Balance zwischen Aktivierung und Schonung, fehlende Berücksichtigung der Umweltfaktoren nach der Internationalen Klassifikation der Funktionsfähigkeit, Behinderung und Gesundheit (ICF) und schwankende psychophysische Leistungsfähigkeit angesehen. Führungskräfte bzw. Arbeitsgeber zeigen diesbezüglich fehlendes Wissen [34]. Es bieten sich hier Leistungen zu Teilhabe am Arbeitsleben, den sog. LTAs, an [34], die beantragt werden können.

Die SARS-CoV-2-Pandemie stellt sich als eine besondere Herausforderung der Chirurgie mit fachspezifisch immanent intensiverem Patientenkontakt bzw. des gesamten Gesundheitswesens dar, die durchaus teils langanhaltende Veränderungen verursachte und deren adäquate gesundheitsbetreuerische wie auch versicherungsrechtliche Aufarbeitung der (fallspezifischen) Konsequenzen noch beträchtliche Anstrengungen und Ressourcen erfordern dürfte.
